# Integration of photoperiodic and temperature cues by the circadian clock to regulate insect seasonal adaptations

**DOI:** 10.1007/s00359-023-01667-1

**Published:** 2023-08-16

**Authors:** Sergio Hidalgo, Joanna C. Chiu

**Affiliations:** Department of Entomology and Nematology, College of Agricultural and Environmental Sciences, University of California, Davis, CA 95616 USA

**Keywords:** Seasonal adaptations, *Drosophila melanogaster*, Circadian clock, Splicing, Temperature, Neuropeptide

## Abstract

Organisms adapt to unfavorable seasonal conditions to survive. These seasonal adaptations rely on the correct interpretation of environmental cues such as photoperiod, and temperature. Genetic studies in several organisms, including the genetic powerhouse *Drosophila melanogaster*, indicate that circadian clock components, such as *period* and *timeless*, are involved in photoperiodic-dependent seasonal adaptations, but our understanding of this process is far from complete. In particular, the role of temperature as a key factor to complement photoperiodic response is not well understood. The development of new sequencing technologies has proven extremely useful in understanding the plastic changes that the clock and other cellular components undergo in different environmental conditions, including changes in gene expression and alternative splicing. This article discusses the integration of photoperiod and temperature for seasonal biology as well as downstream molecular and cellular pathways involved in the regulation of physiological adaptations that occur with changing seasons. We focus our discussion on the current understanding of the involvement of the molecular clock and the circadian clock neuronal circuits in these adaptations in *D. melanogaster*.

## Introduction

Animals adapt their physiology and behavior in anticipation of seasonal changes in environmental conditions (Denlinger 2022; Lincoln 2019). These seasonal adaptations offer a survival advantage and vary greatly across species. Some animals, for example, engage in long migrations (Alerstam and Bäckman 2018; Chowdhury et al. 2021; Reppert et al. 2016) while others enter prolonged states of developmental arrest (i.e., diapause) (Denlinger 2022, 2023). Although the nature of these adaptations may seem very different, they all rely on the successful organismal interpretation of environmental cues.

In temperate regions of the planet, daylength (i.e., photoperiod) serves as a universal signal for these adaptations largely due to its reliability and marked difference across seasons (Saunders 2020; Saunders and Bertossa 2011). For overwintering animals, for instance, late summer and early autumn present shorter and shorter days, and these photoperiodic changes are responsible for triggering seasonal adaptations. Nonetheless, seasonal adaptations are known to occur in organisms living in tropical regions with little to no photoperiodic changes (Dani and Sheeba 2022; Denlinger 1986; Pollock et al. 2019; Wenda et al. 2023). This begs the question of which other environmental cues are used to produce and regulate seasonal adaptations.

The ability of organisms to integrate and interpret seasonal cues has been discussed for almost a century. In 1936, Erwin Bünning proposed that one of the functions of the circadian clock, the mechanism responsible for maintaining daily rhythms, is working as a photoperiodic timer (Bünning 1936, 1960). Shortly after, and following Bünning’s ideas, Colin Pittendrigh pioneered work in insects to support an involvement of the circadian clock in photoperiodism (Pittendrigh et al. 1958; Pittendrigh and Minis 1964). Since then, much of our understanding of the molecular and neuronal basis of photoperiodism, and the role of the circadian clock in this process, comes from extensive work conducted on several insects including the bean bug *Riptortus pedestris* (Hasebe and Shiga 2021, 2022; Ikeno et al. 2014; Koide et al. 2021; Shimokawa et al. 2008), the flesh fly *Sarcophaga argyrostoma *(Saunders 1971, 1973, 1975), and in several *Drosophila* species (Breda et al. 2020; Collins et al. 2004; Kyriacou et al. 2008; Majercak et al. 1999; Menegazzi et al. 2017; Pittendrigh 1954; Saunders et al. 1989; Saunders and Gilbert 1990; Schiesari et al. 2011; Shearer et al. 2016).

In this review, we discuss our current understanding of the molecular pathways and neuronal circuits associated with seasonal adaptations, with a special focus on the observations made in *D. melanogaster*. We also discuss our understanding of how photoperiod and temperature are integrated by the circadian clock, and how this could induce seasonal adaptations.

## Diapause or quiescence?

An important distinction has been observed regarding the nature of seasonal adaptations that animals experience in response to photoperiod. Diapause can be defined as a programmed developmental arrest that can be irrespective to the environmental conditions (i.e., obligatory diapause) or can occur in response to a sustained change in environmental conditions, namely photoperiod (i.e., facultative diapause). Regarding the latter, some animals seem to have a strong facultative diapause response at high temperatures of around 20 °C, like *S. argyrostoma* (Saunders 1973) or the white butterfly *Pieris brassicae* that can diapause at even higher temperatures of around 28 °C (Bünning and Joerrens 1962). Others, like *D. melanogaster*, require temperatures as low as 12 °C to trigger this process (Saunders and Gilbert 1990). This marked difference in temperature requirement has led to redefining the diapause response of *D. melanogaster* as quiescence instead (Denlinger 2023; Lirakis et al. 2018; Saunders 2020).

**Fig. 1:**
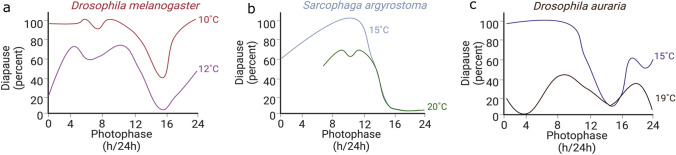
The effect of temperature on daylength-dependent diapause incidence in insects. Diapause incidence across different photophases is shown for **a** *Drosophila melanogaster* at 10 °C (red line) and at 12 °C (purple line), **b** for *Sarcophaga argyrostoma* at 15 °C (grey line) and at 20 °C (green line), and **c** for *Drosophila auraria* at 15 °C (blue line) and at 19 °C (black line). The degree to which temperature and day-length affect diapause incidence is species-specific. Adapted from Minami et al. (1979), Saunders (1971)  and Saunders et al. (1989)

Organisms that undergo diapause have a critical daylength or critical photoperiod, which is the photoperiod at which 50% of the animals in a given population enter into diapause. In contrast, quiescence is an immediate reaction to changes in conditions, such as temperature. Descriptive studies in the late 90s by Saunders and Gilbert (1990) showed the sensitive nature of the photoperiodic response in *D. melanogaster*. Under cold temperatures (< 12 °C), the effect of photoperiod is almost negligible and almost all females enter reproductive dormancy, which is characterized by the predominance of pre-vitellogenic, underdeveloped eggs. Some groups have argued that this response is akin to quiescence or better profiled as a stress response (Lirakis et al. 2018). Although this conclusion is tempting, the effect of photoperiod itself cannot be ignored. With increasing temperatures up to 12 °C, the proportion of females exhibiting reproductive dormancy considerably decreases, however, the critical photoperiod is still visible in photoperiods of around 12–14 h of light (Fig. 1a). Furthermore, a study using constant, low temperature and long photoperiod have shown that *D. melanogaster* fails to enter diapause, as evidenced by the presence of big ovaries with developed eggs (Abrieux et al. 2020). Finally, there are many shared characteristics of quiescence in *D. melanogaster* with diapause in other species that show stronger photoperiodic control, including stress resistance and increased lipid storage (Kubrak et al. 2014; Schmidt and Paaby 2008; Sinclair and Marshall 2018). For these reasons, we will continue our d

iscussion below by referring quiescence in *Drosophila* species as a type of diapause response that shows high sensitivity to temperature.

Interestingly, the modulation of the diapause response by temperature is observed across many animals, but the extent varies greatly. In the flesh fly *S. argyrostoma*, for example, diapause is achieved at temperatures as high as 20 °C, and decreasing the temperature to 15 °C increases the amplitude of the response, meaning the peak percentage of diapause at a given critical daylength, without changing the critical daylength (Fig. 1b) (Saunders 1971). Considering that these temperatures are often encountered in nature, it is possible that both signals are interpreted by the seasonal timer to regulate diapause. Moreover, in some insects, photoperiod seems to be irrelevant. Rather, it is the exposure to different temperatures that induces overwintering, like in the cabbage beetle *Colaphellus bowringi* (Wang et al. 2007; Xue et al. 2002).

The idea that both photoperiod and temperature contribute to seasonal adaptations is not new (Saunders 2014). Several studies showed that small variations in temperature can have an immense effect on diapause incidence. In *D. melanogaster*, both amplitude of the diapause response (i.e., percentage of the population entering diapause) and critical daylength are greatly affected by temperature, with little diapause occurring at 13 °C and almost complete diapause at short days at 10 °C; this is a minor difference of only 3 °C (Saunders and Gilbert 1990). A similar sensitivity is displayed by the fly *Drosophila auraria* (Fig. 1c) and the knotgrass moth *Acronicta rumicis* that shortens its critical daylength with lower temperatures (Kimura 1990; Minami et al. 1979; Saunders and Gilbert 1990). In contrast, the white butterfly *P. brassicae* shows remarkable temperature-compensation across a wide range of temperatures such that diapause can be induced at temperatures as high 28 °C (Bünning and Joerrens 1962). Therefore, the effect of temperature on the photoperiodic timer seems to be rather variable across species.

It is possible that in some geographical regions, this sensitivity difference might be of significant importance. For instance, tropical regions show little to no variation in photoperiod across the calendar year, yet seasonal breeding patterns and other adaptations are still widespread (Denlinger 1986). As little photoperiodic information is available to distinguish seasons in these regions, small variations of temperature and humidity could have a huge impact on diapause incidence. For instance, a difference of 3°–5° can be observed across monthly average temperatures throughout the calendar year, which seems to be enough to modulate some insects’ diapause response, as discussed above (Denlinger 1986, 2023). Thus, differences in thermal sensitivity observed across species could be a function of geographical localization as well as local adaptations. *D. melanogaster* is considered to be of tropical origin (Lachaise et al. 1988); thus its photoperiodic response might be a more recent event that is highly impacted by a more variable, less predictable, and ancient temperature-driven timer. This also explains the recent polymorphisms that have not achieved fixation in some *D. melanogaster* populations across temperate regions like *l-tim and ls-tim* alleles that affect photoperiodic responses, as we will discuss later (Tauber et al. 2007; Trotta et al. 2006). Conversely, other species that originated in temperate regions, like *P. brassicae*, evolved to have robust temperature compensation in a seasonal timer highly driven by photoperiodism (Spieth and Cordes 2012).

Given these observations, it is likely that temperature might serve as a seasonal cue in conjunction with photoperiod and that temperature sensitivity is a function of the geographical origin of the species, giving a special preponderance to photoperiodic cues in temperate regions (Hut et al. 2013; Trotta et al. 2006). This is clear when comparing the clinal effect on diapause incidence across different populations of *D. melanogaster* (Pegoraro et al. 2017; Schmidt et al. 2005; Schmidt and Paaby 2008) and other species (Han and Denlinger 2009; Saunders 2021; Yamada and Yamamoto 2011), including the seminal work of Lankinen (1986, 1993) and Takamura and Pittendrigh (Pittendrigh et al. 1991; Pittendrigh and Takamura 1989). It seems that both temperature and photoperiod act as either complementary or alternative environmental inputs to regulate seasonal adaptations. In this case, it would be appropriate to re-evaluate the definitions of quiescence in *D. melanogaster* as diapause, but dependent on both temperature and photoperiod. But even if we consider this idea as a possibility, it is still unclear how these cues are integrated within the organism.

## Modified coincidence model

The external coincidence model proposed by Bünning and refined by Pittendrigh and others (Pittendrigh 1972; Saunders 1978) suggests that a photosensitive process laying in the scotophase (dark phase) during short days, coincides with the photophase in long days, inducing development and preventing diapause (Pittendrigh 1966). On the other hand, the internal coincidence model suggests the existence of two independent oscillators that track dawn and dusk, and that the phase relationship between these two, regulated by photoperiod, determines entry or exit to overwintering processes (Pittendrigh 1972). Both models have proved to be useful in explaining the functioning of the seasonal timer in some species. However, in some species like *D. melanogaster*, which also relies on temperature, these models seem to be insufficient as changes in solely photoperiod at high temperatures are unable to regulate diapause.

**Fig. 2:**
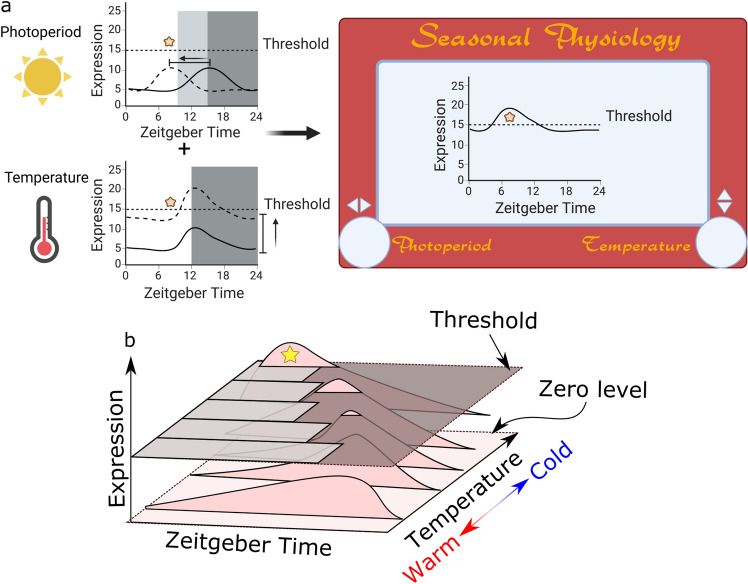
The influence of temperature on the photoperiodic control of diapause. **a** The top graph depicts the effect of photoperiod on the peak phase of a clock-regulated protein (Protein X) that exhibits daily oscillation in expression level. Dark grey depicts dark period for long days and light grey and dark grey together depict dark period for short days. The solid line represents daily protein expression in long days while the dotted line denotes expression in short days. The protein oscillation in short days peaks at a time that is in phase required for a developmental landmark, in this case a diapause-inducing event (marked by the yellow star symbol). However, the insect does not enter diapause given the protein level has not reached a required threshold for diapause induction. The lower graph depicts temperature-dependent modulation of the median of this daily protein oscillation, i.e. modulation of overall expression without changing the oscillation. The integration of photoperiodic and temperature effects allows the peak of daily expression to surpass a hypothetical threshold, but the phase of the peak does not coincide with the diapause-inducing event. The concurrence of both the optimal photoperiod and diapause-inducing temperature produces the overlap of the peak of the daily oscillation to the diapause-inducing event permitting initiation of diapause (right panel). The nature of this axial modulation can be represented as the 1960’s kids toy Etch-A-Sketch. **b** If integrated into the external or internal coincidence model, it is appropriate to think of temperature as another axis. A 3-axis rendition of the model presented in **a** shows the course of change of the oscillation of Protein X required to generate diapause-inducing events (depicted by yellow star). As the season progress, a change from long days (day: light gray, night: dark gray shown on top of graph in 3D) to short days, with a decrease in temperature

Taking this into account, a modified coincidence model can be proposed in which the photosensitive process, a diapause-inducing or developmental landmark in the external coincidence model, is positioned over a threshold that is set depending on the species. For instance, let us consider the oscillation of a protein (Protein X) whose expression at a particular time-of-day (phase) and at a particular level is required to trigger a developmental landmark (e.g., diapause initiation; Fig. 2; yellow star). For this to occur, the integration of two independent processes is essential: a change in the phase of the daily oscillation and a change in the overall levels of this protein. In the case of phase, changes in photoperiod might be sufficient to trigger advancement or delay in the phase at which the protein level is at its daily peak, (Fig. 2a; upper left panel). Now, as discussed before, changes in photoperiod are not enough for some species to trigger diapause. In that regard, we propose that temperature acts as an additional signal to modulate the overall levels of this protein leading it to surpass a threshold (Fig. 2a; lower left panel). Note that the change triggered by temperature can be either at the midline (i.e., the average or middle of the sinusoidal daily oscillation), meaning that the overall levels of the protein are increased without changes in the amplitude of the oscillation (as depicted in Fig. 2a, lower left panel) or with changes in the amplitude without change in the overall levels (i.e., same baseline). As separate cues, photoperiod and temperature might not be sufficient to reach the developmental landmark; however, together they are able to tune the expression of Protein X to reach the required developmental mark (Fig. 2a; right panel). Although we present this model using diapause initiation as the developmental landmark, this model can also be easily adapted for development initiation after exit from diapause.

This axial translation of protein expression, modulated by two environmental cues, temperature and photoperiod, can be represented by the popular 1960s kids’ toy Etch-A-Sketch, in which the turns of two knobs moves a stylus horizontally and vertically, generating lines along the x and y axis (Fig. 2a; right panel). There are some examples in *D. melanogaster* where elements of the circadian clock and clock-controlled genes respond in this fashion. For instance, the amplitude of the oscillation of the clock gene *period* (*per*) increases with colder temperatures, while changing the photoperiod from 12 h light:12 h dark cycles (12:12 LD) to 6:18 LD renders an advancement in the phase of *per* oscillation of about 6 h (Majercak et al. 1999). Additionally, we showed that changing the photoperiod from 16:8 LD to 8:16 LD causes an 8-hour delay in the peak of the protein EYES ABSENT (EYA), whose expression is key to triggering reproductive dormancy (describe in more detail later), from the middle of the day to the middle of the night. Consistent with the model, decreasing the temperature to 10 °C alone generates an increase in the overall levels of EYA with marginal change in its phase (Abrieux et al. 2020). Finally, we recently showed that the clock output peptide Pigment Dispersing Factor (PDF) also responds to photoperiod and temperature albeit in an opposite manner to the one we described for EYA. This led us to hypothesize and show that PDF could be upstream of EYA to regulate seasonal adaptations (Hidalgo et al. 2023).

It is important to note that our model does not conflict with either the external or internal coincidence model. Instead, our proposal takes into account a third axis, i.e., temperature, that can be applied to either model regardless (Fig. 2b). Investigating the role of temperature in photoperiodism might be important to uncover new mechanisms in which the clock participates in seasonal timing. This model might prove useful when analyzing the difference between different populations of a single species across different latitudes. For instance, this can be applied to explain the differences in temperature sensitivity, as measured by developmental times, in *D. melanogaster* populations expressing different alleles of the *timeless* gene (Andreatta et al. 2023).

### Molecular and cellular mechanisms regulating seasonal adaptations

#### The role of the molecular circadian clock on photoperiodism

The first clear indications of the involvement of the circadian clock in photoperiodism came from experimental observations and protocols such as the Nanda-Hamner protocol (Nanda and Hamner 1958) (reviewed in Teets and Meuti 2021). In this protocol, researchers keep organisms at different conditions varying only the length of the nights with a fixed length of the light phase. If the clock mediates photoperiodism, an increase in short-day phenotype (i.e., diapause incidence) is expected to appear once the length of the night reaches a multiple of 24 h. This approach was key to suggesting a possible role of the circadian clock in photoperiodism in *D. melanogaster* (Saunders 1990). Since then, the bloom of the genetic era and access to modern genetic manipulation techniques have allowed us to investigate the specific role that molecular clock components play in seasonal adaptations, if any.

Circadian clocks in animals and plants rely on transcriptional-translational feedback mechanisms to regulate endogenous 24-hour rhythms (Patke et al. 2020). Key transcriptional activators promote the expression of clock-controlled genes, including the expression of genes that encode transcriptional repressors that feedback to negatively regulate the activators to maintain self-sustaining molecular rhythms. In *D. melanogaster*, the activators are *clock* (*clk*) and *cycle* (*cyc*), and the repressors are *period* (*per*) and *timeless* (*tim*) (Allada et al. 1998; Hamblen-Coyle et al. 1989; Hardin et al. 1990; Rothenfluh et al. 2000; Sehgal et al. 1994). A light-responsive intracellular photoreceptor CRYPTOCHROME (CRY), encoded by the *cry* gene, is responsible for light-mediated TIM degradation, which is critical for photoentrainment (Busza et al. 2004; Emery et al. 1998; Koh et al. 2006; Stanewsky et al. 1998). *per, clk*, and *cyc* are highly conserved between species, while some elements were substituted due to gene duplication and loss (Lam and Chiu 2019). For instance, CRY was replaced by a light-insensitive CRY (mammalian CRY; *m-*CRY) that performs the function of TIM in several species including bees and humans (Cai and Chiu 2021; Goto 2022).

**Fig. 3:**
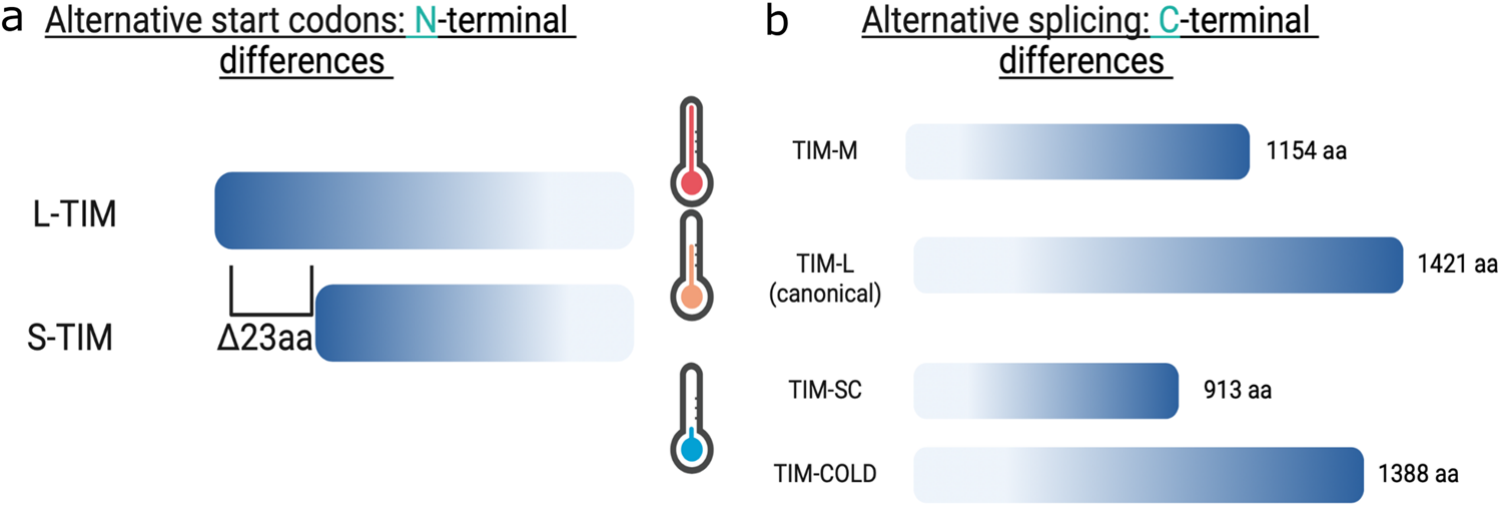
Different isoforms of clock gene timeless. **a** Alternative codon usage at the N terminal gives rise to two possible TIMELESS proteins from the *ls-tim* alleles differing in 23 amino acids. **b** Thermosensitive splicing gives rise to four different TIMELESS variants; TIM-MEDIUM in response to warm temperatures (TIM-M; Shakhmantsir et al. 2018), the canonical TIM-LONG (TIM-LONG), and TIM-SHORT COLD (TIM-SC) and TIM-COLD at cold temperatures (Abrieux et al. 2020; Martin Anduaga et al. 2019). The specific roles of these isoforms in diapause incidence have not been functionally tested. Note that the “L” isoforms generated from N-terminal differences are not the same as the ones described for the C-terminal, L-TIM and TIM-L, respectively

The contribution of molecular clock proteins to seasonal adaptations has been assessed extensively by genetic association studies. In *D. melanogaster*, diapause incidence varies by latitude, suggesting differential allele selections across different populations (Schmidt et al. 2005; Schmidt and Paaby 2008). Consistent with this, and highlighting the involvement of the circadian clock in diapause inducibility, two *tim* alleles were detected in different fly populations in Italy: *ls-tim* and *s-tim* (Sandrelli et al. 2007; Tauber et al. 2007). The “*ls*” allele produces a full-length mRNA of 1421 nucleotides (L-TIM) and a shorter variant of 1398 nucleotides (S-TIM), while the “*s*” allele produces only the shorter variant S-TIM (Rosato et al. 1997a, b) (Fig. 3a). The *ls-tim* allele correlates with higher levels of diapause in Europe while the *s-tim* does not (Zonato et al. 2018), and the *s-tim* allele is associated with temperature-dependent decrease in developmental time and increased egg production (Andreatta et al. 2023). Clinal variation was also observed in other clock components, including *per.* The *per* gene has a varying number of threonine-glycine repeats, and the frequency of the allele containing 20 repeats changes across latitudes (Costa et al. 1992; Costa and Kyriacou 1998; Rosato et al. 1997a, b). Changes in the number of repeats have been associated with temperature compensation of the circadian clock, highlighting the role of this gene in the relationship between the clock and temperature. Studies in flesh-fly *Sarcophaga bullata* showed that non-diapausing strains have higher expression of *per*, suggesting a role of this gene in seasonality (Goto et al. 2006). Additionally, a higher incidence of diapause was observed in strains carrying a shorter PER C-terminal region, further supporting a role of these genes in seasonality (Han and Denlinger 2009).

Functional relationships between these genetic variations and the seasonal timer have also been investigated but yielded conflicting results. In 1989, Saunders et al. utilized the *per* mutants generated by the Benzer lab to directly test the influence of the circadian clock on diapause incidence (Saunders et al. 1989). The results of these experiments were ground-breaking. Four different *per* mutants were able to enter photoperiodic-dependent diapause at 12 °C, albeit with a different critical daylength. Further studies suggested that *per* is probably not important for photoperiodism or has a limited impact on this process (Emerson et al. 2009; Saunders 1990). This indicated, in principle, that the circadian clock might not be important for seasonality. Nonetheless, other studies showed that *per* is important in other photoperiodic-dependent traits in *D. melanogaster* as *per* mutants lose photoperiodic-dependent cold tolerance (Pegoraro et al. 2014). This apparent conflict on the role of *per* in photoperiodism in *D. melanogaster* highlights the complexity of the traits tested in this species. It is possible though that diapause and cold tolerance are two different outputs of seasonal adaptations that are modulated by different mechanisms. In this case, *per* would not be a fundamental gene for photoperiodism per se, instead, relevant in the downstream process of cold tolerance with no effect on diapause.

Evidence for the role of *per* in photoperiodism in other organisms is much stronger. In the bean bug *R. pedestris*, knocking down *per* promotes development, even under diapause-inducing conditions (Ikeno et al. 2010, 2011). A similar result was observed in the parasitoid wasps *Nasonia vitripennis*, in which a reduction in *per* expression produces females that remain in the active reproductive state even under short days (Mukai and Goto 2016). Similarly, work in the domestic silk moth *Bombyx mori* showed that knocking out *per* prevents response to short days (Ikeda et al. 2021; Tobita and Kiuchi 2022), and knocking down *per* by double-stranded (ds) RNA in the mosquito *Culex pipiens* prevents adaptations to short photoperiod, including inhibiting diapause and inhibiting an increase in lipid storage (Meuti et al. 2015). Now, with all this information, it is safe to say that *per* is an important regulator of diapause in several insects. What remains unclear, however, is whether the role of *per* is due to a pleiotropic effect independent of its circadian clock function or whether it is a direct effect of the clock, as a modular entity for seasonality.

In *D. melanogaster*, *tim* null-mutants lose photoperiodic-dependent development of cold tolerance, similar to *per* mutants (Pegoraro et al. 2014). Moreover, the same mutant show non-diapausing phenotypes even under short photoperiod at cold temperatures, as assayed by ovary size, while overexpression of *tim* generates small ovaries even during long days (Abrieux et al. 2020). In the cabbage beetle *C. bowringi* knocking down *tim* results in impaired lipid storage (Zhu et al. 2019), common in non-diapausing individuals, similar to what happens while knocking down *tim* in *Cx. pipiens* (Meuti et al. 2015). Thus, the negative elements of the clock, and potentially the clock itself, act as a module that seems to be indispensable for a correct photoperiodic response. Yet, there is still the issue as to whether temperature response is an integral part of the circadian clock, and by this definition, of the seasonal adaptation machinery.

#### The role of the molecular circadian clock on temperature integration: splicing as a driving force of seasonal adaptations

As discussed above, temperature seems to be a key factor necessary for inducing photoperiodic responses in *D. melanogaster*. Temperature on its own modulates *D. melanogaster* locomotor activity, an effect of temperature-dependent *per* splicing (Chen et al. 2007; Majercak et al. 1999; Zhang et al. 2018). Under 12:12 LD cycles at 25 °C, *D. melanogaster* shows two well-defined peaks of activity: a morning peak and an evening peak. When temperature drops to 18 °C or lower, the evening peak of activity advances around 4 h into midday (Hidalgo et al. 2023; Majercak et al. 1999). As an ectotherm, *D. melanogaster* adjusts its body temperature with the help of environmental temperature, driven by temperature preference (Hamada et al. 2008). Thus, this change in locomotor activity is believed to be a seasonal adaptation that promotes activity during the light phase at low temperatures. Associated with this change, an increase in *per* mRNA is observed at cold temperatures, produced by an increase in the splicing of an intron in the 3′ end of the *per* transcript (Chen et al. 2007; Majercak et al. 1999). As a result, the accumulation of PER and TIM proteins occurs earlier, modulating the locomotor rhythms downstream.

Several thermosensitive splicing events also occur resulting in different *tim* isoforms (Fig. 3). Under cold conditions, an isoform that is 33 amino acids shorter than full-length TIM (TIM-L; not to be confused with L-TIM produced by N-terminus variations discussed above) is produced, termed TIM-cold (Boothroyd et al. 2007) (Fig. 3b). Both *per* and *tim* splicing events are also observed under natural conditions with peak unspliced *per* and spliced *tim* observed in cold months from October through March (Montelli et al. 2015). Another *tim* isoform, derived from an intron retention event that causes cleavage and polyadenylation of a short isoform termed *tim-sc*, was also recently described (Abrieux et al. 2020; Martin Anduaga et al. 2019; Shakhmantsir et al. 2018) (Fig. 3b). Functional studies of this isoform are still being conducted. Martin Anduaga et al. (2019) showed that the overexpression of this isoform in a *tim-*null background modulates the evening peak of locomotor activity in a similar fashion as *per* splicing but less pronounced. Hence, it is possible that these splicing events work in concert to modulate seasonal adaptations, including locomotor adaptations.

It is interesting that many of these changes have a high impact on the structure of clock components. For instance, the difference in the proteins L-TIM and S-TIM produced by the *ls-tim* and *s-tim* alleles is about 23 amino acids, which is sufficient to modulate the interaction between TIM and CRY (Montelli et al. 2015). L-TIM has reduced interaction with CRY that results in reduced light sensitivity. If we now consider the possible splicing events of these alleles, a staggering number of combinations of TIM isoforms are possible. Montelli et al. (2015) addressed this issue by testing CRY interaction to L-TIM and S-TIM in its spliced and unspliced form (the splicing event that gives rise to *tim-cold* and not *tim-sc*) using a yeast two-hybrid system. The unspliced S-TIM had a higher affinity for CRY compared to the other combinations. Likewise, unspliced S-TIM had a higher binding affinity to PER, especially during the dark phase. Interestingly, this in vitro approach seems to be functionally relevant, as it shows that reduced light sensitivity of L-TIM enables *D. melanogaster* in northern Europe to adapt to long days in the summer (Deppisch et al. 2022; Lamaze et al. 2022). On the other hand, it is not known what the functional consequences are when other TIM isoforms are expressed within the molecular clock, particularly the functional consequence of a shorter TIM (i.e., TIM-SC; Fig. 3b). Considering that the cytoplasmatic localization domain and a fragment of the second PER binding domain are missing, it is expected that subcellular localization and even the interaction of TIM-SC and PER would be different (Cai and Chiu 2021). This is also important considering that under cold conditions, the *tim-sc* isoform is predominant with little to no expression of the full-length canonical *tim* (Abrieux et al. 2020; Martin Anduaga et al. 2019). Future research focusing on the functional consequences of these structural changes on the clock protein behavior is going to provide answers to these pressing questions.

### From neuropeptides to hormones: integration of seasonal cues and downstream pathways

Seasonal adaptations come with a wide array of physiological adaptations that rely on the regulation of hormonal changes. In *D. melanogaster*, these changes seem to start in a group of dorsal medial neurosecretory cells, called insulin-producing cells (IPCs) (Schiesari et al. 2011; Sim and Denlinger 2013). These cells release insulin-like peptides (DILPS) down the recurrent nerve to the corpus allatum (CA) and the corpus cardiacum (CC) (Nässel and Zandawala 2020). The IPCs are required for regulating diapause given that the ablation of these cells enhances diapause (Schiesari et al. 2016). Additionally, hyperactivating or reducing the electrical excitability of the IPCs prevents or induces diapause, respectively (Schiesari et al. 2016). Consistent with this, overexpression of DILP2-5 peptides prevents diapause at 12 °C and short days while flies lacking *dilp1-5* or *2*, *3*, and *5* have increased diapause incidence even after being transferred to higher temperatures (Schiesari et al. 2016). Double mutants for *dilp2-3* and *dilp5* have stronger diapause induction compared to control flies, further confirming the role of DILP in diapause inhibition (Kubrak et al. 2014). Nonetheless, contrary to what would be expected, independent studies showed that levels of *dilp1*, *2*, *3*, and *5* mRNAs are increased instead of decreased in diapausing flies (Kubrak et al. 2014; Liu et al. 2016; Schiesari et al. 2016). The nature of this paradox is not well understood. It has been suggested that this could be part of a feedback mechanism in which a reduced activity/function of DILPS under diapause-inducing conditions triggers an increase in the mRNA levels of these peptides (Schiesari et al. 2016) or that this state of hormonal imbalance could correspond to a new homeostatic state in which DILPS and other hormones, such as the adipokinetic hormone (Akh), are working concertedly to modulate metabolism (Kubrak et al. 2014). Nonetheless, as the author in the later study suggested, changes in *dilp* expression are not a direct indication of the release of the peptides. It is possible that the levels of the peptides (protein) are still low in diapause-inducing conditions despite the high levels of transcripts (mRNA). This is consistent with increased FOXO transcriptional activity, measured as a readout of the reduced DILP signaling, in diapausing flies (Schiesari et al. 2016). More studies are required to clarify this issue. Yet from functional studies, it is possible to suggest that under diapause-inducing conditions, reduced activity of the IPCs potentially could reduce the secretion of DILPs. The reduction of circulating DILPs in turn decreases the activation of the CA, consequently reducing the release of juvenile hormone (JH), a hormone that is key for vitellogenesis and that is involved in diapause (Kurogi et al. 2021; Saunders et al. 1990). The IPCs do not express a functional clock (Barber et al. 2016; Cavanaugh et al. 2014), therefore, it is believed that the integration of seasonal cues occurs upstream, in the circadian clock neuronal network, and then relayed to these neurosecretory cells.

**Fig. 4:**
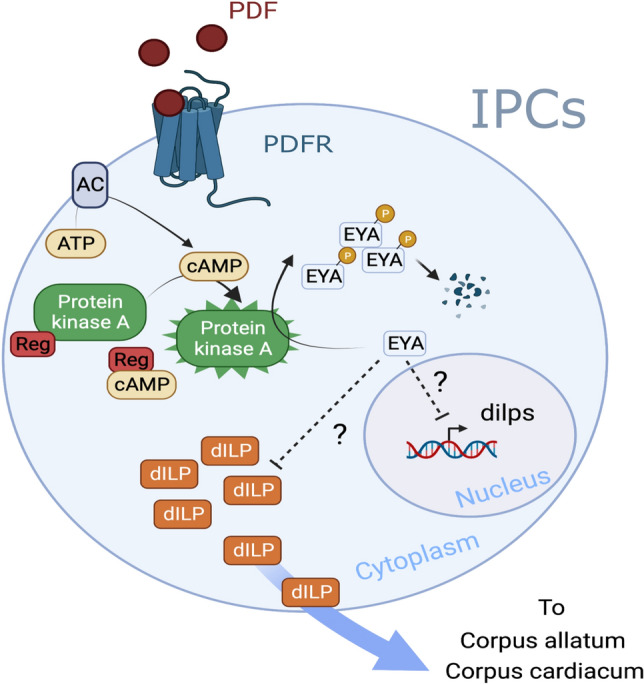
Molecular basis of seasonal control in *Drosophila melanogaster* insulin-producing cells (IPCs). Upon the arrival of the peptide Pigment Dispersing Factor (PDF) to the IPCs, an increase in cAMP and activation of Protein Kinase A (PKA) lead to the phosphorylation and therefore degradation of the protein EYES ABSENT (EYA). EYA promotes diapause potentially by transcriptional regulation of *Drosophila* insulin-like peptides (dILPs) that normally reach the corpus allatum and corpus cardiacum to induce ovarian development and control of lipid storage (Hidalgo et al. 2023; Kubrak et al. 2014; Schiesari et al. 2016)

The circadian clock neuronal network is composed of ~ 150 neurons organized in dorsal neurons (DN1anterior; DN1a, DN1posterior; DN1p, DN2, DN3), dorsal-lateral neurons (LNd), ventral-lateral neurons (LNvs), and lateral posterior neurons (LPN) (Beer and Helfrich-Förster 2020). These neurons form an interconnected network that signals through the co-transmission of small neurotransmitters and neuropeptides (Crespo-Flores and Barber 2022; Duhart et al. 2020; Fujiwara et al. 2018; Goda et al. 2019; Hamasaka et al. 2007; Kunst et al. 2014; Reinhard et al. 2022; Shafer et al. 2008; Yao and Shafer 2014; reviewed in Nässel 2018). Of these clusters, a group of LNvs called the small LNvs (s-LNvs) are involved in diapause control. Activation of the s-LNvs prevents diapause even in dormancy inducing conditions, making this cluster a candidate for seasonal integration (Nagy et al. 2019). Additionally, the s-LNvs express the peptide pigment dispersing factor (PDF) and the short neuropeptide F (sNPF) (Nässel 2018), both of which were shown to be important for diapause as overexpression of either one in the LNvs caused a reduction in diapause incidence (Nagy et al. 2019). The s-LNvs directly signal to the IPCs, an effect that is mediated by the PDF receptor, a G-protein coupled receptor that increases cAMP levels upon activation (Lear et al. 2005; Nagy et al. 2019), highlighting the role of these peptides and the LNvs in seasonal control. Additionally, the exact mechanism by which the circadian clock, through PDF, regulates the hormonal cascade under diapause conditions is still unclear. Recently, we showed that expression of EYES ABSENT (EYA), a co-transcription factor and phosphatase, in the IPCs promotes reproductive dormancy (Abrieux et al. 2020). EYA level increases under diapause-inducing conditions. Importantly, overexpressing or reducing *eya* in the IPCs promotes or inhibits diapause, respectively. The opposite effect of EYA and PDF on diapause control suggests PDF negatively regulate EYA function. We showed that PDF reduces EYA level through a phosphorylation-dependent regulation mediated by the activation of PDFR and PKA function (Hidalgo et al. 2023) (Fig. 4). Thus, under diapause-inducing conditions, a reduction of PDF would allow EYA accumulation in the IPCs, which then triggers reproductive dormancy. Indeed, PDF levels are responsive to both photoperiod and temperature, providing seasonal integration through a circadian output. On warm and long days, PDF levels in the s-LNvs dorsal terminals, the ones contacting the IPCs, are significantly higher compared to short and cold days (Hidalgo et al. 2023). These low levels in winter-like conditions are explained by a reduction in *pdf* mRNA, which can be subtly observed in *D. melanogaster* at 18 °C in 3’ RNA-seq datasets (Martin Anduaga et al. 2019) and clearly in *Drosophila suzukii* at 10 °C using RNA-seq (Shearer et al. 2016). Although it is still unclear how this is achieved, it is possible that the changes in the molecular clock explained above serve as a conduit to reduce *pdf* during winter, but this needs to be investigated in future studies. Moreover, it is unclear exactly how EYA is connected to the insulin pathway.

The LNvs and PDF have also been associated to the change in locomotor activity under different seasonal conditions. As mentioned before, under cold conditions, there is an advancement of the evening peak of activity rhythm, regulated by *per* and *tim* splicing, an advancement that can be also observed in *Pdf* null mutants and in *pdfr* mutants at 25 °C (Lear et al. 2005; Majercak et al. 1999; Renn et al. 1999). This, in addition to the fact that we observed a reduction in PDF levels under cold conditions, also supports the notion that changes in the molecular clock might drive changes in PDF, ultimately triggering seasonal adaptations (Hidalgo et al. 2023). Interestingly, PDF also serves as a key signal to regulate the delay of the evening peak under long day, warm days (Lear et al. 2009; Vaze and Helfrich-Förster 2021). This is mediated by l-LNvs and the s-LNvs, in contact with the LNds (Schlichting et al. 2016, 2019). Overall, these lines of evidence highlight the role of PDF in responding to seasonal cues, potentially offering a link between the circadian clock and seasonal adaptations in *D. melanogaster.*

It is important to note that the s-LNvs/PDF/IPCs axis is probably not the only pathway to modulate reproductive dormancy. The neuropeptide allatostatin-C (AstC), expressed in DN1p, DN3, and LPN clusters has been shown to participate in circadian control and seasonality. Under warm conditions, DN3s are active and have high AstC levels, while the opposite is observed in the cold (Meiselman et al. 2022). Activation of these neurons promotes egg production, an output of reproductive state, even under cold conditions. Thus, AstC expression in the DN3 cluster is required to inhibit diapause in a temperature-dependent manner, independent of the IPCs and through undetermined cholinergic neurons expressing the AstC receptor R2, one of two AstC receptors described in *D. melanogaster* (Kreienkamp et al. 2002; Meiselman et al. 2022). This seems to be a parallel pathway for regulating egg development as AstC released from the DN1p was shown to inhibit oogenesis through the decrease of DILP2 in the IPCs and a consequential reduction of JH, opposite to the reported role of PDF (Zhang et al. 2021, 2022). Interestingly, another IPC-independent pathway for modulating diapause in *D. melanogaster* has been recently uncovered involving midbrain neurons expressing the Diuretic hormone 31 peptide (DH31), important in daily temperature preference rhythms among other functions (Goda et al. 2016, 2019; Kurogi et al. 2023). These neurons make direct contact with the CA, suppressing the production of JH under winter-like conditions, thus promoting reproductive arrest (Kurogi et al. 2023). Connectomic data showed that these neurons connect with circadian clock neurons, including the s-LNvs, potentially allowing the circadian clock to convey seasonal cues. It appears that direct and indirect pathways (i.e., through the IPCs) work concertedly to trigger seasonal adaptations, with upstream regulation by the circadian clock.

**Fig. 5:**
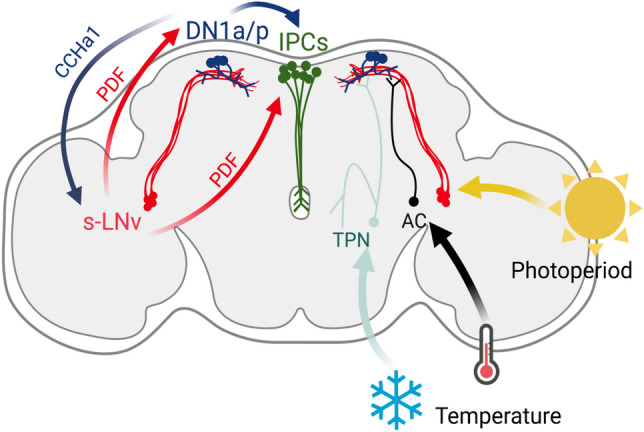
*Drosophila melanogaster* neuronal pathways associated with the integration of seasonal cues. The small ventral lateral neurons (s-LNvs) receive photoperiodic information while the anterior and posterior dorsal neuron 1 (DN1a and DN1p, respectively) cell clusters receive thermal cues from the thermosensitive projection neurons (TPN) and the internal thermosensory anterior cells (AC) (Alpert et al. 2020, 2022; Hidalgo et al. 2023; Schichting et al. 2016, 2019; Jin et al. 2021). The s-LNvs release PDF, a circadian neuropeptide that signals to the DN1a, DN1p, and the IPCs (Im and Taghert 2010; Shafer et al. 2008; Yoshii et al. 2009). The DN1s reciprocally connects with the s-LNvs and controls PDF levels by CCHamide1 (CCHa1) action and signal directly to the IPCs (Barber et al. 2016; Fujiwara et al. 2018). Both s-LNvs and DN1 reciprocally interact to modulate circadian rhythms (Kuwano et al. 2023) and potentially seasonal adaptations through direct action over the IPCs

The circuits that integrate temperature into the brain relay this information to a few clock neuronal cell clusters (George and Stanewsky 2021). The absolute cold and hot temperatures are perceived by thermoreceptors in the antennae (Gallio et al. 2011; Liu et al. 2015) and the chordotonal organs in the legs (Chen et al. 2015; Sehadova et al. 2009). This information travels through thermosensitive receptor neurons that form hot and cold adjacent glomeruli in the posterior antennal lobe (Frank et al. 2015). Thermal cues are then integrated and transferred by thermosensitive projection neurons (Alpert et al. 2020, 2022) and by internal thermosensitive neurons (Hamada et al. 2008) to clock neuron clusters LPN, DN1a, and DN1p. The integration of thermal cues into the circadian clock suggests that seasonal cues, i.e., light and temperature, need to be pre-processed before conveying the information to the IPCs. The DN1a and DN1p neurons are modulated by PDF signaling (Im and Taghert 2010; Shafer et al. 2008; Yoshii et al. 2009) and project to the IPCs directly, where they can drive rhythms in their firing patterns in response to starvation (Barber et al. 2016). Additionally, DN1p neurons have been involved in conveying thermal inputs to promote wakefulness (Jin et al. 2021). Therefore, it is possible that temperature is conveyed to the IPCs through DN1a and/or DN1p with the input of PDF for photoperiodic signals. Importantly, DN1a neurons produce CCHamide1 (Fujiwara et al. 2018; Nässel 2018), a neuropeptide that modulates PDF in the s-LNvs, and DN1p are connected to PDF neurons to modulate adaptations to light intensity (Chatterjee et al. 2018; Kuwano et al. 2023). Thus, it is possible that reciprocal connections between DN1a/DN1p cell clusters and the s-LNvs are required before reaching the IPCs (Fig. 5). It seems evident that these two circadian neuropeptides, PDF and CCHamide1, and possibly others, could work in a concerted action to modulate seasonality through the integration of photoperiod and temperature. Hence, understanding the interaction between the circadian control of peptides and the regulation of the IPCs and other neurosecretory cells will be key to untangling the circadian basis of seasonal physiology.

## Concluding remarks

Seasonal adaptations are key for survival, but the exact molecular and neuronal underpinnings driving these adaptations are still under investigation. The use of genetic approaches has increased our understanding of photoperiodism, but more research is still needed. Although the photoperiodic timer is considered temperature-compensated, the small contribution of temperature to the critical photoperiod depends on the species and populations within those species. It is important to note that the predominant effect of temperature on the termination of diapause (Hodek 2002), which is likely mediated by changes in the clock components, was not discussed in this review. Thus, special care is needed when investigating diapause entry or exit.

Evidence gathered throughout the years suggests a modular contribution of the circadian clock in photoperiodism, supporting Bünning’s almost 100-year-old idea. The differential splicing of clock components adds an exquisite layer of complexity to the control of photoperiodism by the circadian clock. Therefore, the advancement in long-read sequencing technologies is certainly a catalyzing development to further our understanding of this process. Is it possible that other components of the clock, apart from *tim* and *per*, are affected by thermal- or light-sensitive splicing? Or is this process directed to just a few key genes? Future work in the field may reveal whether splicing is an integral process required for seasonal adaptations.

Finally, a large part of our understanding of seasonal adaptations comes from investigating variations observed by animals in temperate zones, which is just a fraction of the cases. Adaptation to seasons on tropical species is also prevalent, but our understanding of how the seasonal timer works at these latitudes is lacking. Future studies investigating a wider range of species will be the key to establishing general principles regarding the interplay between the circadian clock, photoperiod, and temperature regulation.
